# Cellular and Molecular Mechanisms of Functional Hierarchy of Pacemaker Clusters in the Sinoatrial Node: New Insights into Sick Sinus Syndrome

**DOI:** 10.3390/jcdd8040043

**Published:** 2021-04-13

**Authors:** Di Lang, Alexey V. Glukhov

**Affiliations:** Department of Medicine, School of Medicine and Public Health, University of Wisconsin-Madison, Madison, WI 53705, USA; dlang@medicine.wisc.edu

**Keywords:** sinoatrial node, pacemaker cluster, pacemaker shift, ion channel, calcium, sick sinus syndrome

## Abstract

The sinoatrial node (SAN), the primary pacemaker of the heart, consists of a heterogeneous population of specialized cardiac myocytes that can spontaneously produce action potentials, generating the rhythm of the heart and coordinating heart contractions. Spontaneous beating can be observed from very early embryonic stage and under a series of genetic programing, the complex heterogeneous SAN cells are formed with specific biomarker proteins and generate robust automaticity. The SAN is capable to adjust its pacemaking rate in response to environmental and autonomic changes to regulate the heart’s performance and maintain physiological needs of the body. Importantly, the origin of the action potential in the SAN is not static, but rather dynamically changes according to the prevailing conditions. Changes in the heart rate are associated with a shift of the leading pacemaker location within the SAN and accompanied by alterations in P wave morphology and PQ interval on ECG. Pacemaker shift occurs in response to different interventions: neurohormonal modulation, cardiac glycosides, pharmacological agents, mechanical stretch, a change in temperature, and a change in extracellular electrolyte concentrations. It was linked with the presence of distinct anatomically and functionally defined intranodal pacemaker clusters that are responsible for the generation of the heart rhythm at different rates. Recent studies indicate that on the cellular level, different pacemaker clusters rely on a complex interplay between the calcium (referred to local subsarcolemmal Ca^2+^ releases generated by the sarcoplasmic reticulum via ryanodine receptors) and voltage (referred to sarcolemmal electrogenic proteins) components of so-called “coupled clock pacemaker system” that is used to describe a complex mechanism of SAN pacemaking. In this review, we examine the structural, functional, and molecular evidence for hierarchical pacemaker clustering within the SAN. We also demonstrate the unique molecular signatures of intranodal pacemaker clusters, highlighting their importance for physiological rhythm regulation as well as their role in the development of SAN dysfunction, also known as sick sinus syndrome.

## 1. Introduction

The sinoatrial node (SAN) is the primary pacemaker of the heart, which can spontaneously produce electrical impulses coordinating heart contractions. Anatomical SAN is located at the junction where the superior vena cava enters the right atrium [1]. Despite the species difference in size, the SAN covers a relatively large area rather than a small group of cells (SAN cells, or SANCs) where the electrical impulses originate. SAN is bordered from the crista terminalis and may extend from the superior to inferior vena cava. Across this area, the SAN consists of highly heterogeneous populations of cells that significantly vary in size, ionic current and gap junction repertoire, and expression profiles of other biomarkers [2]. At the center of the SAN, a group of small spindle-shaped cells, that can spontaneously generate electrical impulses, is extensively studied and traditionally recognized as “typical nodal cells” or leading pacemaker cells. However, emerging evidence have suggested that they are not the only group of SANCs with automaticity. It was recognized that the origin of electrical impulses is not static or limited exclusively to center SAN, rather, it has been shown that pacemaker location is dynamic and changes according to the prevailing conditions [3], including neurohormonal modulation, pharmacological interventions, mechanical stretch, and a change in temperature among others. Importantly, such pacemaker shift is not arbitrary but rather associated with distinct anatomical regions within the SAN, also known as pacemaker clusters [3,4,5,6]. These intranodal pacemaker clusters can be activated and dominate the heart beating under certain conditions [6,7,8]. It was proposed that any substantial changes in heart rate are associated with the pacemaker shift within the SAN, and larger changes in heart rate were linked with greater distance of pacemaker shift [6]. Thereby, a complex and dynamic system of intranodal pacemaker clusters, which, individually, are responsible for narrow ranges of beating rates, can represent a hierarchical rhythmic system tightly adjusted to regulate the heart’s performance and meet the physiological needs of the body. When activated during physiological stimulations, pacemaker clusters follow a hierarchical way to take turns in determining the heart beating at the exact rate to meet body needs and assures the robustness of heart rhythm maintenance. Importantly, multiple studies have shown that center and peripheral SAN pacemaker clusters possess diverse functional and molecular signatures and presumably can be activated via distinct cellular mechanisms [7,9,10]. In this review, we summarize the evidence supporting the hierarchy of SAN pacemaking system and examine the structural, functional, and molecular evidence for the pacemaker clustering within the SAN. We also demonstrate the unique molecular signatures of intranodal pacemaker clusters highlighting their importance for physiological rhythm regulation as well as for the development of SAN dysfunction, also known as sick sinus syndrome.

## 2. Development of the SAN

Extensive studies have been performed to explore the embryonic origin of the pacemaker tissue [11,12,13]. The clear elucidation of the mechanisms underlying SAN development would extend our understanding of SAN physiology and regulation, and thereby provide foundation for therapeutic intervention of pacemaker diseases. Moreover, the studies of genetic programing that controls SAN development have been critical in the regenerative medicine where nodal-like pluripotent stem cells are preferentially generated [14] and selected [15], which were then used to develop the concept of “biological pacemakers” [16]. In reviewing the development of SAN, we also observe evidence that may support the hierarchy of pacemaker clusters. 

### 2.1. Structural Changes in the SAN Development 

During SAN development, spontaneous contractions can be detected at early stages [11,12,13]. Caudal pacemaker activity conducts slowly in the early heart tube resulting in a sinusoidal morphology ECG [13] and the cells of the early embryonic heart tube present similar properties to mature SAN before the tube elongates and develops into ventricular and atrial chambers. SAN primordium was observed in histological sections as early as embryonic day E10.5 in mice [17], and the nodal structure then becomes obvious in the right sinus horn at the junction with the atrium. A transient development of a small SAN follows [18], which then develop to a full SAN in case of atrial right isomerism [19].

### 2.2. Genetic Programing Controlling SAN Development

A series of specific biomarkers has been reported in identification and localization of sinoatrial precursors in its development. One widely accepted specific biomarker of SAN is the hyperpolarization-activated cyclic nucleotide-gated (HCN) channels, which mediate the *I_f_* current known to gradually depolarize membrane potential during diastolic phase and is essential to SAN automaticity. Among all the isoforms, HCN4 is reported to be the highest expressed one in the adult SAN in different species including human [20,21,22]. HCN4 expression was reported in cells as early as the gastrulation stage (E6.0) in mice [23] and mRNA expression of HCN4 is also detected in the cardiac crescent at E7.5 in mice [24], suggesting that HCN4 is a specific marker that can be used to characterize pacemaker cells at very early stage of the development. However, HCN4 does not seem to be functioning until a later stage as evidenced by the observations that HCN4 knockout mice die between E9.5 to E11.5, but not as early as it was observed in cells [25].

Besides HCN4, there are also other markers that facilitate researchers to study and identify SAN during its development despite less specificity. In the adult rabbit heart, neurofilament-M (NF-M) transcript is found to be localized in all the cardiac condition system, including the SAN, but not the working cardiomyocytes. NF-M mRNA can be detected at E9.5 which also helps to locate SAN origin during the development [26]. Moreover, gap junctions, though not sufficiently expressed during early embryonic stages, are also markers to differentiate the SANCs from surrounding atrial cells. Gap junction protein, connexin 43 (Cx43) which form high conductance junctions between atrial and ventricular cells, is negligibly expressed in the SAN [27,28]. Similarly, the gene for atrial natriuretic peptide, natriuretic peptides A (*Nppa*), shows low expression in the SAN unlike in atrial cells.

During the development of the SAN, the genetic program for pacemaker properties is promoted while the genetic program that promotes chamber specification is inhibited. There are a series of transcription factors that were reported to be fundamental in such genetic program [28]. For example, T-box transcription factors, are known to be critical in maintaining the pacemaker features during SAN development. Tbx3 is found to be specifically expressed in the regions that eventually form the mature conduction system of the heart [29]. The lack of Tbx3 can induce SANCs to express specific genes of the working myocardium including Cx43 and nppa [30]. Similar to Tbx3, Shox2 (short-stature homeobox protein 2) is also reported to facilitate the maintenance of SAN properties during development. Shox2 is a transcriptional repressor with its expression restricted to the right side of the sinus venosus, where it forms the SAN later in development. Knockdown of Shox2 causes the ectopic expressions atrial genes like Cx43 and Nppa and lack of of Tbx3 and HCN4 [31]. Tbx18 was also identified to be an important factor in the formation of SAN head during development [32]. Mice lacking Tbx18 are found to have smaller SAN, which is attributed to delayed recruitment of SAN progenitors [32]. ISL1 (Islet-1) is also found to be critical in the functioning of pacemaker cells during development. In an SAN-specific *Isl1* deletion model, it is revealed that a number of critical genes for SAN function including L-type Ca^2+^ channel, Tbx3 and Ank2 are downstream of ISL1 activity [33]. 

Other transcription factors, including Tbx5 and Nkx2.5, on the other hand, promote the expression of these specific genes in working myocytes and therefore are considered as negative markers of the SAN. Studies also showed that deficiency of Nkx2.5 promotes the atrial myocytes to express SAN phenotype proteins including HCN4 and Tbx3 [18]. Overexpression of Nkx2.5 in the atrium on the other hand, causes the expression of atrial genes. Shox2, which promotes SAN gene expression, is reported to repress the Nkx2.5 gene [31]. The genetic programing is very complex, where tiny interventions in this cascade may result in cells not with SAN phenotypes, but with both atrial and SAN characteristics [18,30].

It is established that a conserved gene regulatory network of these transcription factors controls the development and function of sinus node cells. A recent study which employs the an epigenetic assay for transposase-accessible chromatin with sequencing further investigated the precise mechanism that connect the expression of these regulators with their gene targets and found that sinus node cells have distinct regions of accessible chromatin that correlate with their gene expression profile and contain novel SAN enhancers during development [34]. 

## 3. Concept of Hierarchical Pacemaker Clustering in the SAN

### 3.1. Pacemaker Shift

In early histological studies, the SAN was described as a small condensed area of specialized tissue located near the crista terminalis where the superior vena cava enters the right atrium [35]. Recent studies in canine [36], rabbit [1] and human [37] hearts, however, suggested that SAN is a more diffusive and extensive structure than it was appreciated previously. Both functional and structural assessments have shown that the SAN region covers a relatively large region extending from the superior to inferior vena cava, with a indistinct and irregular margin to its surrounding atrial myocytes [38]. Within this region, highly heterogeneous populations of cells with varying morphologies and properties (see Section 4 below) are observed. In 1985, Kreitner isolated the inter-caval region, including the SAN and peripheral atrium, from the rabbit atria and cut it into several small strips. He found that multiple (but not all) strips, including those from the SAN center, near the SAN periphery and even from adjacent atria, possess spontaneous automatic activity [39]. The author concluded that, in addition to typical nodal cells, there are other pacemaker cells that occupy a relatively large area around the SAN and can generate spontaneous electrical impulses when they are physically (or electrically) uncoupled from the center of the SAN.

**Figure 1:**
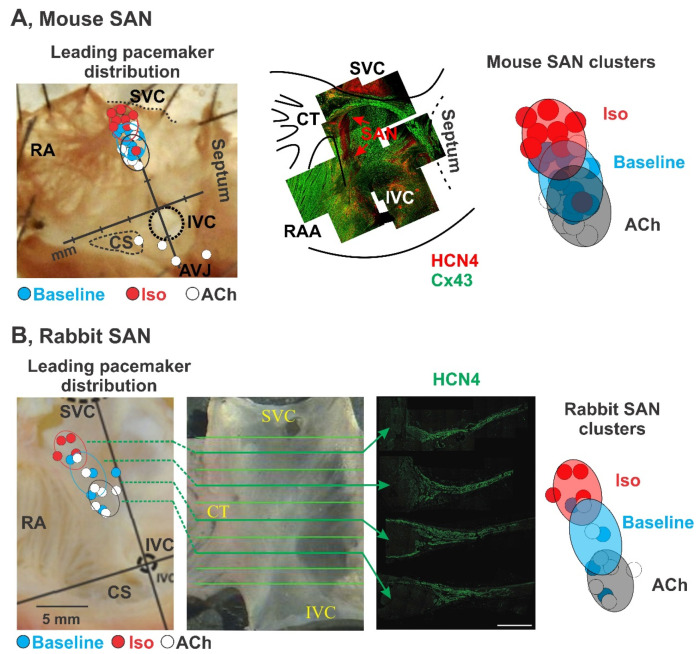
Pacemaker shift in mouse and rabbit SANs. Figures are modified from Glukhov [48,49] for mouse, and Lang [7], for rabbit. (**A**) Pacemaker shift in the mouse SAN under sympathetic (isoproterenol, or Iso; red dots) and parasympathetic (acetylcholine, or ACh; white dots) stimulations. Leading pacemakers under different conditions were visualized by optical mapping in isolated mouse right atrial preparations. SAN area was identified by connexin 43 (Cx43)-negative and HCN4-positive immunofluorescent staining. Iso shifted the leading pacemakers superiorly and ACh shifted them inferiorly within the anatomically and functionally defined SAN. (**B**) Pacemaker shift in rabbit SAN under sympathetic (Iso; red dots) and parasympathetic (ACh; white dots) stimulations. Leading pacemakers distributed within three distinct areas of the SAN, which is identified as HCN4-positive area. Similar to mouse, Iso and ACh shifted the leading pacemakers from the center of the rabbit SAN to superior and inferior intranodal pacemaker clusters, respectively.

**Figure 2:**
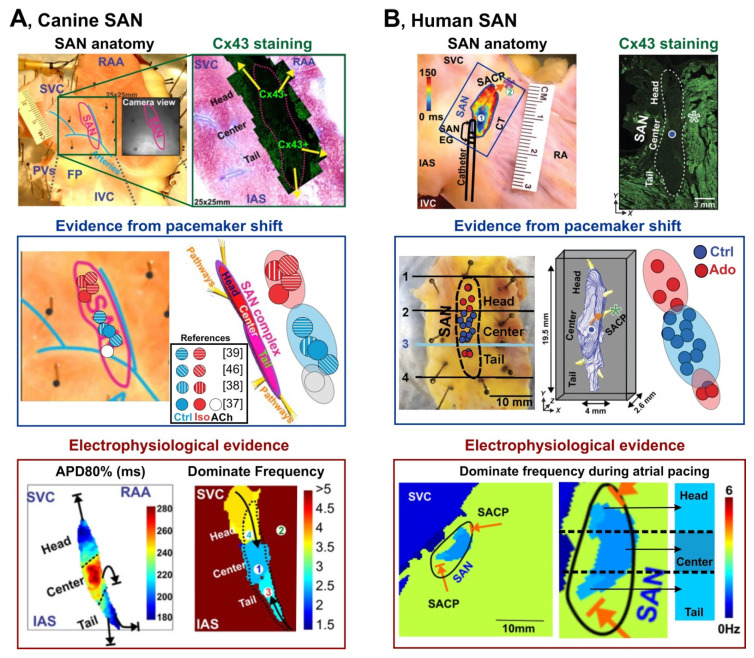
Evidence of several pacemaker clusters in canine and human SANs. Figures are modified from Lou [50] and Li [4]. (**A**) Evidence supporting existence of pacemaker clusters in the canine SAN. Three pacemaker clusters located at head, center and tail SAN were identified during autonomic stimulation. Similar to mouse and rabbit SANs, Iso and ACh shifted the leading pacemaker from the center cluster (blue dots in a light blue area) into superior (red dots in a light red area, Iso) and inferior (white dots in a gray area, ACh) clusters. The figure summarizes four studies that optically located the leading pacemaker in the canine SAN [42,43,44,51]. Immunofluorescent detection showed various Cx43 expression patterns in distinct intranodal clusters. Further evidence from electrophysiological characterization also supported the existence of pacemaker clusters located at head, center and tail SAN, which is consistent with the findings from the pacemaker shift. Three clusters showed different action potential duration (APD) and dominate frequency distribution measured during atrial pacing. (**B**) Evidence supporting existence of multiple pacemakers in the human SAN. Similar to the canine SAN, three pacemaker clusters were identified in the human SAN evidenced from pacemaker shift and dominant frequency distribution during atrial pacing. Adenosine (Ado) shifted the leading pacemaker outside the central SAN majorly into the superior cluster and rarely to the inferior cluster. Green *: sinus exit pathway.

Later studies confirmed that indeed such “non-typical nodal cells” with intrinsic automaticity belong to the SAN and can serve as a leading pacemaker under certain conditions. In a series of studies, the location of the leading pacemaker was found to be highly variable and not limited to a certain anatomical area (i.e., the central SAN) [7,40,41,42,43,44,45]. It was shown that the leading pacemaker can change its location within the anatomically and structurally defined SAN region which was described as a “pacemaker shift” [46]. The leading pacemaker can shift both superiorly, inferiorly, or laterally in response to various physiological stimulations. Sympathetic stimulation shifts the leading pacemaker superiorly as labeled by red dots, and parasympathetic stimulation shifts the leading pacemaker inferiorly as labeled by whit dots in mouse, rabbit (Figure 1), and canine (Figure 2). Importantly, pacemaker shift is accompanied by changes in SAN beating rate. Pacemaker shift also alters the activation sequence of the atria and thereby could be observed from the changes of ECG P wave morphology and polarity [47]. 

Importantly, the anatomical sites that the leading pacemaker shifts to is consistent between multiple species under various conditions [7,42,52]. Figure 1 and Figure 2 summarize the location of the leading pacemaker observed under baseline conditions and during sympathetic and parasympathetic stimulations in a series of studies in isolated mouse [48], rabbit [7], canine [42,43,44,45,50,51], and human [4] SANs. In those studies, location of the leading pacemaker was accurately captured by a high-resolution optical mapping [53,54] based on the earliest impulse origination [55,56]. Sympathetic stimulation was found to shift the leading pacemaker location superiorly in every individual animal to confined locations shown by red circles. Similar, parasympathetic simulation resulted in an inferior shift as shown by white circles. Importantly, all the leading pacemakers, identified both at baseline and under autonomic stimulation, were located within the Cx43 negative and HCN4 positive region that confirms their localization within the SAN. Furthermore, under specific conditions, the leading pacemaker locations are concentrated within distinct “clusters”. Such clusters could be attributed to certain populations of pacemaker cells that dominate under certain physiological conditions and generate a faster pacemaking rate comparing to baseline condition resulting in a concomitant pacemaker shift. In addition to the pacemaker shift, evidence from studies from canine and human have further supported the existence of multi intranodal pacemaker clusters. Several groups have independently reported the superior and inferior pacemaker shift during anatomical stimulation in canine (Figure 2A, middle panel) [42,43,44,45]. Immunofluorescent studies showed different Cx43 expression within these superior and inferior pacemaker clusters (Figure 2A, top panel) [42]. Moreover, distinct electrophysiological parameters could also differentiate the multi intranodal pacemaker cluster as shown in Figure 2A, bottom panel. Specifically, three pacemaker clusters located at SAN head, center and tail have different action potential durations [42]. These clusters also present different dominant frequencies during fast atrial pacing. Similarly, in the human heart, adenosine was found to shift the leading pacemaker to both the SAN head and tail (Figure 2B, middle panel), and, as reported in the canine SAN, the dominant frequencies in these three pacemaker clusters during atrial pacing were also different (Figure 2B, bottom panel) [4]. 

**Figure 3:**
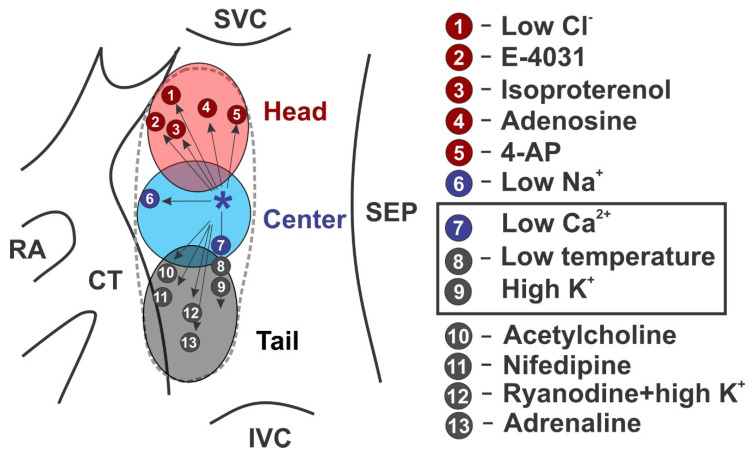
Summary of the pacemaker shifts among multiple SAN clusters. Figure is modified based on the data from Boyett et al. [5] with adding data from Lang et al. (point 3) [7], Li et al. (point 4) [4] and Shinohara et al. (point 13) [43]. All points are from rabbit SAN experiments except otherwise indicated. Diagram summarizes pacemaker shift into different clusters under the following conditions. *—pacemaker at baseline condition. Shift to the superior pacemaker cluster: 1—low Cl^-^ concentration; 2—treatment with E-4031 (class III antiarrhythmic drug that blocks hERG K^+^ channels); 3—isoproterenol (non-selective β-adrenergic agonist); 4—adenosine (human data), and 5—4-aminopyridine (4-AP, selective blocker of *I*_to_ current). Shift within the center pacemaker cluster: 6—low Na^+^ concentration; 7—low Ca^2+^ concentration, low temperature, or high K^+^ concentration. Shift to the inferior pacemaker cluster: 8,9—same condition as for 7, but to different extend. Shift to the inferior pacemaker cluster: 10—acetylcholine (ACh, muscarinic receptor agonist); 11—nifedipine (Ca^2+^ channel blocker); 12—ryanodine with high extracellular K^+^ concentration (canine data); and 13—adrenaline (α- and β-adrenergic receptor agonist).

Pacemaker shift could be also observed in various physiological conditions. Besides autonomic stimulation, cardiac glycosides [57], L-type Ca^2+^ channel blocker nifedipine [58], ryanodine treatment [43], and changes in temperature [41] were all observed to shift the leading pacemaker to clusters located at different anatomical regions within the SAN, as summarized in Figure 3.

### 3.2. Dynamic Hierarchy of Cluster Pacemaking 

Pacemaker shift is always accompanied by a change in beating rate, which seems to be closely correlated with the location the leading pacemaker moves to, or in other words, the beating rate seems to be closely correlated with distinct pacemaker clusters [7,9]. Shibata et al. found that vagal stimulation results in the shift of the leading pacemaker to a “lower” (inferior) position in accordance with a slowing of the beating rate. Importantly, the authors noticed that the extent of rate slowing is proportional to the distance of the pacemaker shift. They found the greater the slowing, the larger distance of the pacemaker shift [6]. In the pacemaker shift path, multiple pacemaker clusters were dominating at different beating rates, with each cluster working within a narrow frequency window [6,59]. The pacemaker shifts appear to follow a dynamic hierarchical pattern, in which another pacemaker is activated and dominate at rhythms that exceed the current pacemaker’s working range. This dynamic hierarchical pacemaker shift is supposed to be mediated by a combination of complex factors discussed in Section 4.

The intrinsic automaticity of each pacemaker cluster is not static but rather dynamic and regulatable in response to prevailing conditions. In a recent study, Lang et al. used optical mapping of the rabbit SAN to identify and isolate intranodal pacemaker clusters that dominated at baseline and sympathetic stimulation. The cluster that dominated under sympathetic tone showed no automaticity at baseline condition; whereas, after application of 100 nM isoproterenol, the cluster’s pacemaking activity dramatically increased. Under sympathetic stimulation, the pacemaker cluster which dominated at baseline condition have also showed an increase in pacemaking rate which, however, was not as fast as that in other clusters. The baseline pacemaker then lost its dominating role which resulted in a pacemaker shift to another cluster activated by sympathetic stimulation [10]. This study suggests that each intranodal pacemaker cluster can be specifically activated to start providing the dominant rhythm.

Besides adjusting the heart rates by shifting to distinct pacemaker clusters, the hierarchical pacemaking system could also provide specific mechanisms preventing rhythm failure. Li and colleagues found that in humans, distinct intranodal pacemaker clusters work in a hierarchical and complementary pattern that ensures a “fail-safe” mechanism for robust maintenance of sinus rhythm [4]. During adenosine stimulation, when the primary central SAN pacemaker cluster is suppressed, other clusters (superior and inferior), which were previously inactive, take over and maintain a robust heart rhythm. This cluster system works synergistically with the sinoatrial conduction pathways to rescue sinus rhythm when needed [4,60,61]. When preferential sinoatrial conduction pathways were shown to be suppressed together with the inactivation of certain pacemaker clusters under conditions like adenosine stimulation, another sinoatrial conduction pathway could be activated along with change of pacemaker clusters.

### 3.3. Synchronization of the Hierarchical Pacemaker Clusters

With distinct intrinsic automaticity of each pacemaker cluster, the SAN synchronizes all of them and generates an “integral” rhythm. This rhythm is mainly determined by the cluster that displays the fastest firing rate [62]. Pacemaker clusters are connected through low resistance gap junctions, and affect each other by local circuit currents, which is known as mutual entrainment [63,64]. The mutual entrainment is associated with the respective intrinsic frequencies of the pacemaker clusters, their phase relations and the electrical coupling degrees among them, and eventually synchronizes all the pacemaker cells [63]. In a recent study by Fenske and colleagues, a nonfiring mode of pacemaker cells was introduced [62]. Pacemaker cells can be hyperpolarized to a degree that prevents its own spontaneous firing, i.e., nonfiring mode. This mode is specifically regulated by the cyclic adenosine monophosphate (cAMP)-dependent regulation of the HCN4 channels [62]. The interactions between nonfiring and firing pacemaker cells form the tonic adjustment of heart rate via synchronization of these two types of pacemaker cells. Specifically, pacemaker cells, that hyperpolarize themselves to enter nonfiring mode, will activate tonic flows of cations from the neighbor firing cells via gap junctions. The current then depolarizes the nonfiring cells and results in a relatively slow firing rate in these cells instead of nonfiring mode. Meanwhile, the source firing cells would be slightly hyperpolarized during this process and fire more slowly, therefore adjusting the SAN to fire at a slower heart rate.

## 4. Functional and Molecular Signatures of SAN Pacemaker Clusters

### 4.1. Fine Architecture

Substantial heterogeneity of pacemaker cells within the SAN has been demonstrated in various studies [9,10,65,66]. It was shown that SANCs significantly vary in their spontaneous beating rate [9,65], cell size [1,66,67,68], cellular microarchitecture [5,69,70], ionic current densities [9,65,70], ratios between depolarizing and repolarizing ionic current [5,9,65,71,72], and cell response to autonomic modulation [9]. SANCs show a distinct cell morphology as reviewed in detail by Boyett et al. [5]. In general, SANCs are smaller and lack of organized myofilaments and mitochondria (cells look “empty” under the light microscope) as compared to the surrounding atrial cells, with an approximately twice higher density of nuclei than in the atrial muscle. James et al. described human and canine SANCs as P. cells as they appear pale in contrast to working myocardium, many of their features resemble those of embryonal primitive myocardial cells, and they also have many similarities to Purkinje cells [68]. Interestingly, the authors noticed that most of the central portion of the SAN is composed of clusters of P. cells, but the homogeneity of the node varies in different areas. The cells in the center of the SAN are reported to be 5–10 μm in diameter in the human and dog [68] as well as in other species [5], compared to 15–25 μm atrial myocytes [73] and even larger ventricular myocytes [74]. Furthermore, several authors observed an increase in cell size and density with more myofilament organization and mitochondria in the SAN periphery [1,59,66,67].

### 4.2. Electrophysiological Heterogeneity

Several groups tried to correlate the SANC size with electrophysiological characteristics, including electrical activity and ion channel composition. Honjo et al. reported in the rabbit SAN that the action potential amplitude, maximum diastolic potential, take-off potential, and action potential upstroke velocity were greater in larger SANC which correlated with a faster spontaneous beating rate [65]. The authors linked this to a higher density of *I*_f_ current and the presence of tetrodotoxin-sensitive *I*_Na_ current found in larger SANCs which are believed to be located in the periphery of the SAN (see above). In contrast, Monfredi et al. recently used 150 spontaneously beating rabbit SANCs (versus 61 cells in Honjo et. study) to study correlations between cell size, spontaneous beating rate and ion current densities, and found no relationship between beating rate and cell size, but also observed a positive correlation between the density of *I*_f_ current (105 cells versus 12 cells in Honjo et. study) and spontaneous beating rate [9]. The authors also observed a strong negative correlation between the spontaneous beating rate and the density of *I*_Ca,L_ current, i.e., those cells that beat the quickest at baseline tend to have the lowest density of *I*_Ca,L_, in contrast to Honjo et. study where no correlation between *I*_Ca,L_ and beating rate was found. Importantly, either study did not attempt geographical categorization of the studies cells or intra-SAN localization of these cells. Therefore, while significant morphological and electrophysiological heterogeneity of SANCs could be appreciate from these studies, it is quite difficult to locate different populations of SANCs to certain intra-nodal pacemaker clusters. However, this could be approximated based on functional behaviors of those SANC populations and their response to various pharmacological interventions.

### 4.3. Pacemaking Mechanisms: Voltage Versus Calcium Clocks

In the study by Monfredi et al., the authors used cyclopiazonic acid (CPA), a moderate disruptor of Ca^2+^ cycling and action potential firing rate in SANCs [75], to determine the effect of Ca^2+^ cycling inhibition on beating rate and the relationship with cell size and ionic current density [9]. It was found that cells with lower *I*_f_ current densities demonstrated a significantly greater susceptibility to the effect of CPA in terms of beating rate slowing. The authors also noted that a relatively large cell population (21 of 90 cells) stopped beating when the sarcoplasmic reticulum pumping rate decreased in the presence of CPA, despite a relatively high *I*_f_ density. It should be noted that in spontaneously beating SANCs, *I*_f_ current density varied dramatically from 0 to ~50 pA/pF, i.e., some of the spontaneously beating SANCs had little to zero *I*_f_. The authors then suggested that those cells may represent a divergent population of SANCs compared with those that simply slow their beating rather than stopping, and thus may rely on pacemaker mechanisms other than *I*_f_, e.g., Ca^2+^ clock. By using computational simulations, Monfredi et al. confirmed that indeed a higher sarcoplasmic reticulum Ca^2+^ pump rate in cells with low *I*_f_ can accelerate diastolic depolarization via increase in *I*_NCX_.

Subsequently, two recent reports from Lakatta’s group demonstrated in guinea pig [76] and human SANCs [77] several populations of cells isolated from the SAN anatomically and functionally defined area which show rhythmic pacemaking activity, dysrhythmic firing, and no spontaneous activity (i.e., “dormant” cells). Dysrhythmic and dormant SANCs have smaller and desynchronized LCR activity than rhythmic SANCs. However, in response to sympathetic stimulation, all dysrhythmic cells and a third of dormant SANCs increased their LCR activity and developed automaticity resulting in spontaneous electrical beating [76]. Importantly, under sympathetic stimulation, dysrhythmic and dormant cells can generate spontaneous activity at the same rate as rhythmic cells at baseline or even faster. This may further support their ability to lead the SAN rhythm under sympathetic stimulation.

Whether or not these different types of SANCs are associated with different pacemaker clusters are responsible for certain heart rates, remains open to question. However, it may provide a mechanistic basis for dynamic pacemaker location shifts within the SAN as it was observed experimentally in intact optically mapped mouse, rabbit, canine, and human SAN preparations (Figure 1 and Figure 2) [4,7,42,48,49,50,52]. As summarized in Figure 1, sympathetic stimulation by isoproterenol in isolated mouse and rabbit SAN preparations results in a shift of the leading pacemaker location superiorly within the anatomically and structurally (identified by connexin 43-negative and HCN4-positive expression patterns) defined SAN area (Figure 1). Therefore, it might be possible to associate SANCs identified by Monfredi et al. as those with lower *I*_f_ current densities which pacemaking greatly relies on the Ca^2+^ clock, with the superior SAN cluster. Indeed, optical mapping of electric activity from isolated superfused rabbit SAN preparation showed that selective inhibition of *I*_f_ current by ivabradine (1–10 μM) resulted in the inferior shift of the leading pacemaker location within the SAN region [78]. These results further highlight a region-specific expression of *I*_f_ current, its contribution to SANC pacemaker activity, and a potentially Ca^2+^ clock driven superior SAN pacemaker cluster.

Concurrently, Li and colleagues performed molecular mapping of HCN isoform expression in human SAN and observed no difference in the expression levels of HCN1, HCN2 and HCN4 between superior, center and inferior SAN pacemaker clusters [79]. However, as different isoforms of the HCN channels exhibit varied sensitivity to cAMP [80], which may differ between intranodal pacemaker clusters, cAMP may have a larger role in regulating SAN pacemaking. In general, SAN is characterized by a significantly higher level of basal cAMP than that found in atrial or ventricular myocytes [81] and appears to be critical for SAN pacemaking. High cAMP concentration is the SAN has been linked to both activation of *I*_f_ current and stimulation of the Ca^2+^ cycling via protein kinase A (PKA)- and Ca^2+^/calmodulin-dependent protein kinase II (CaMKII)-dependent phosphorylation of Ca^2+^ handling proteins, including RyRs, SERCA and phospholamban [62,82,83]. Overexpression of a Ca^2+^-activated adenylyl cyclase, that drives the phosphorylation-driven automaticity, has been shown to be sufficient, in the absence of *I*_f_ current activation, to produce biological pacemaking for 7 days in experimental dogs [84]. Therefore, it is possible that a highly dynamic cAMP cycling profile, rather than HCN expression, determines the shift of the leading pacemaker upon *I*_f_ block.

Recent studies have proposed the structural foundation for cAMP-dependent so-called Ca^2+^ “super-hubs” which have been identified in mouse, rat, rabbit and human atrial myocytes [85,86]. These Ca^2+^ “super-hubs” where linked to specialized axial tubule junctions which are highly enriched by cholesterol-rich caveolae-shaped nanodomains visualized by the fluorescent cholesterol analog dye Chol-PEG-KK114 in live cells. This is different from ventricular myocytes, where transversal (T)-tubules rarely contain caveolae-shaped membrane structures. It was shown that axial tubule junctions form contact junctions with the sarcoplasmic reticulum and are associated with cAMP-dependent highly phosphorylated RyRs [85] which may play an important role in SANC pacemaking in cells located in the periphery of the SAN. Indeed, transmission electron microscopy studies by Ayettey and Navaratnam demonstrated that transversal-axial tubule system is either absent or significantly less developed in the center of the rat SAN [87] but appears in transitional cells within the SAN region. These transitional myocytes resemble nodal cells in diminutiveness of size and lack of atrial granules but also possess a sparse and disorganized transversal-axial tubule system [87]. Overall, these studies highlight the importance of cAMP signaling and Ca^2+^ clock in peripheral SAN clusters.

Indeed, parasympathetic stimulation by acetylcholine perfusion [4,48,49,52] or vagal nerve stimulation [45], which inhibits both voltage and Ca^2+^ clocks, suppresses both center and superior SAN pacemaker clusters and shifts the leading pacemaker location site inferiorly within the SAN area, as summarized in Figure 1 and Figure 2. This may indicate that the inferior SAN pacemaker cluster depends less on Ca^2+^ clock, in general, and cAMP level, in particular. It also may indicate the presence of tetrodotoxin-sensitive *I*_Na_ current as it was identified by Honjo et al. in the periphery of the SAN [65]. More intensive parasympathetic stimulations result in local SAN inexcitability while cells from subsidiary pacemaker areas as well as atrium remain excitable [88,89]. Interestingly, cholinergically induced suppression of action potential amplitude was shown to be predictable based on the maximal rate of action potential upstroke (dV/dt). Vinogradova and colleagues showed in the rabbit SAN that the probability of amplitude suppression was the highest among pacemaker cells (dV/dt, < 3 V/s), in which acetylcholine suppressed amplitude in 93% of cells, and vagal stimulation did so in 81% of cells. With increasing upstroke velocity, the probability of amplitude suppression is significantly decreased and inexcitability did not occur in cells whose dV/dt was >15 V/s. The observed effects were completely reversible and were abolished by atropine, a muscarinic receptor antagonist. These results further support the presence of *I*_Na_ in the peripheral SAN clusters. Indeed, Lipsius and Vassalle [90] and Kreitner [39] observed that in the intact SAN of the guinea-pig and rabbit, tetrodotoxin (a selective *I*_Na_ blocker) reduced the upstroke velocity in the periphery but not in the center. The presence of *I*_Na_ may facilitate spontaneous activity in peripheral SAN cells which primarily rely on Ca^2+^ clock spontaneous local releases from the sarcoplasmic reticulum.

A higher contribution of the Ca^2+^ clock into pacemaking in the periphery of the SAN was supported by immunocytochemical labeling of Ca^2+^ handling proteins, including Ca_v_1.2 isoform of the L-type Ca^2+^ channel as well as NCX1, RyR2, and SERCA2 proteins. Musa and colleagues showed that, in all cases, there was significantly denser labeling of cells from the periphery of the SAN than of cells from the center [70]. The authors demonstrated that a difference in cell size exists between the center and periphery of the SAN, where large cells (C_m_ > 30 pF) tend to be from the periphery and small cells (C_m_ < 30 pF) tend to be from the center of the SAN [70]. In contrast, in the rabbit SAN study by Lyashkov et al., the density of Ca^2+^ machinery proteins were found to be independent of cell size. It should be, however, noted that the authors did not differentiate cells from center or periphery SAN area [91]. Moreover, the studies by Musa et al. and Lyashkov et al. have used different criteria to classify SANC populations. In the study by Musa and colleagues, the authors used two groups of cells. The size of the center SANCs was averagely around 500 µm^2^ and the size of the periphery cells was averagely around 800 µm^2^. In contrast, Lyashkov et al. separated cells into three groups based on their size: 156–262 µm^2^, 324–779 µm^2^ and 806–1187 µm^2^. Thus, the difference between center (~500 µm^2^) and periphery (~800 µm^2^) SAN cells found by Musa et al. might be hidden in the middle group (324–779 µm^2^) of Lyashkov et al. study. The conclusions from two separate studies may not be opposite, but rather the data analysis and interpretations are different.

Intranodal heterogeneity in the expression of repolarizing potassium currents could be also appreciated from the distribution of action potential duration within the SAN. The changes in the action potential are complex and occur in two dimensions. The action potential duration is greatest at or near the leading pacemaker site and it decreases in all directions as demonstrated by Boyett et al. in the rabbit SAN [71]. Similar action potential duration distribution patterns have been shown in the canine SAN as well [50]. Such heterogeneity could be associated with a cluster-specific expression of transient outward K^+^ current *I*_to_ and delayed rectifier K^+^ currents *I*_K,r_ and *I*_K,s_. Boyett et al. showed that inhibition of *I*_to_ by 4-aminopyridine increased the duration of the action potential and the increase was greater in peripheral tissue than in central tissue [71], indicating a greater impact of *I*_to_ in the SAN periphery. The authors also demonstrated a higher impact of 4-aminopyridine-sensitive currents in the inferior region of the SAN than the superior region. In contrast, an opposite pattern was observed for *I*_K,r_. Kodama et al. examined the effect of *I*_K,r_ antagonist E-4031 in the superior and inferior direction within the rabbit SAN and found that the center and inferior SAN clusters were more sensitive to *I*_K,r_ inhibition than the superior cluster [72]. As a result, E-4031 suppressed the pacemaker activity in the center SAN and shifted the leading pacemaker superiorly (Figure 3).

### 4.4. Autonomic Innervation

The rich innervation of the SAN is well documented [92]. Anderson showed that the SAN is highly supplied with plexuses of both acetylcholinesterase- and catecholamine-containing nerves [93]. SAN autonomic control is achieved by a complex interplay between the extra-cardiac neural circuits that interface with specific ganglionated plexus found in epicardial fat pad within the intercaval region at the dorsal aspect of the right atrium [94,95]. As discussed in Section 4.3 above, autonomic stimulation of the SAN is associated with a pacemaker shift within or outside of the SAN. This was attributed to the varying sensitivity of the SAN pacemaker clusters to autonomic stimulation, both sympathetic and parasympathetic. Different sensitivity of the SAN pacemaker clusters to autonomic stimulation, in principle, could be linked to specific ion channel repertoire in distinct clusters, heterogeneous autonomic innervation, or both.

It is believed that a negative chronotropic effect of acetylcholine, released from parasympathetic vagal nerves, on the SAN is mediated by activation of acetylcholine-activated inwardly-rectifying potassium current *I*_K,ACh_, hyperpolarizing shift in the *I*_f_ activation curve, and inhibition of *I*_Ca,L_. While *I*_K,ACh_ activation is achieved through a direct interaction of M_2_ muscarinic receptors with GIRK4 channels, *I*_f_ and *I*_Ca,L_ currents are regulated by changes in cAMP concentration induced via acetylcholine-dependent inhibition of adenylyl cyclase activity. In contrast, a positive chronotropic effect of noradrenalin, released from the sympathetic nerves, on the SAN purely depends on changes in cAMP concentration, associated with β-adrenergic receptor dependent stimulation of adenylyl cyclase, and a subsequent depolarizing shift in the *I*_f_ activation curve and a potentiation of *I*_Ca,L_ and *I*_K_ activities. In addition, studies indicate that cAMP-mediated PKA-dependent modulation of the Ca^2+^ component of the SAN pacemaker system can significantly contribute to autonomic modulation of heart rate [96]. Therefore, different sensitivity of the SAN pacemaker clusters to both parasympathetic and sympathetic stimulations, that was observed experimentally and clinically, could be associated with heterogeneity in the expression levels of both voltage (including *I*_f_, *I*_Ca,L_, and *I*_K_) and Ca^2+^ clock proteins as discussed above. It, however, could be also linked to heterogeneous autonomic innervation of the SAN.

Indeed, different densities of muscarinic and adrenergic receptors as well as different innervation has been proposed in the SAN [97,98]. Beau et al. [98] applied quantitative light-microscopic autoradiography of radioligand binding sites to characterize the spatial distribution of muscarinic cholinergic and β-adrenergic receptor subtypes in the canine SAN. The authors have found that the region of dominant pacemaker activity, localized with in vitro electrical mapping, consistently exhibited greater densities of muscarinic and β_1_-adrenergic receptors than other SAN regions. Muscarinic receptor density in the dominant pacemaker region was 18 ± 2% and 29 ± 7% higher than in adjacent superior and inferior SAN clusters, respectively. Density of β_1_-adrenergic receptors in the dominant site was 53 ± 5% and 26 ± 4% higher than in adjacent superior and inferior SAN regions, respectively. Later study by Kurogouchi et al. in dogs [99] confirmed a higher density of muscarinic receptors in the central SAN pacemaker cluster compared with superior and inferior clusters, but did not find any difference for β-adrenergic receptor subtypes. In contrast, in the rat SAN, Sutyagin and colleagues [100] have shown that the relative density of binding sites for β-adrenergic and muscarinic cholinergic receptors was minimum in the central SAN cluster and asymmetrically increased to maximum values to cranial (sharply) and caudal (smoothly) directions.

These data indicate that pacemaker shift can result from a complex interaction between both intrinsic properties of SAN clusters (including expression levels of voltage and Ca^2+^ clock proteins as well as muscarinic and adrenergic receptors) and heterogeneous innervation of intranodal clusters. Importantly, these changes can be species-dependent and rely on the dynamic balance (i.e., “accentuated antagonism” [101,102]) between the sympathetic and parasympathetic branches of the autonomic nervous system.

Overall, these findings demonstrate substantial heterogeneity of cells within the SAN that could be associated with distinct functional intranodal pacemaker clusters. Importantly, these clusters are characterized by different ion channel and Ca^2+^ cycling protein compositions and, likely, varying contribution of voltage and Ca^2+^ clock components of the coupled-clock system into their pacemaker mechanisms. The latter may be critical in pathological conditions associated with mutations/impairment of certain pacemaker proteins which would affect SAN pacemaking in a cluster-specific manner and thus be manifested differently and upon specific circumstances as discussed in the next section.

## 5. Hierarchical Pacemaker Clusters in Sinus Node Dysfunction

Pacemaker clusters have diverse functional and molecular signatures and thereby can be activated during physiological stimulations and hierarchically take turns to dominate the heart beating with rhythm of exact rates that meets body needs. To generate rhythm at different rates, pacemaker clusters may have distinct pacemaker mechanisms that probably involve a dynamic balance between the components of the coupled clock system. As mentioned earlier, this system integrates molecular cues on the cell membrane surface (i.e., voltage clock) and the intracellular Ca^2+^ machinery proteins (Ca^2+^ clock) to synergistically depolarize membrane potential during diastole and eventually boost it to the threshold for *I*_Ca,L_ activation and initiate spontaneous action potentials.

The diverse molecular foundation of each pacemaker cluster may suggest unequal contribution of pacemaker components from the coupled clock system in the pacemaking, or, some clusters may depend more on membrane clock in its pacemaking, whereas pacemaking in other clusters may be mediated more by the rhythmic Ca^2+^ oscillations. The remodeling of certain pacemaking components then is anticipated to disrupt pacemaker clusters to different extents leading to SAN dysfunctions. SAN dysfunction is a major public health problem that refers to abnormalities in SAN impulse formation and propagation, which has been associated with a series of gene mutations [69]. Since the contribution of each pacemaking component varies between the intranodal clusters, genetic mutations will affect the clusters differently depending on the encoded mutant gene. Therefore, SAN dysfunction could occur only when the affected cluster is supposed to be activated. For example, in patients with catecholaminergic polymorphic ventricular tachycardia (CPVT), a genetic disorder associated with mutations in Ca^2+^ handling proteins, 5–20% are documented with SAN dysfunction [103]. Though the majority of CPVT patients show normal SAN function at rest, researches have shown that the effect of sympathetic and parasympathetic stimulations on heart rate regulation are dramatically attenuated [49,104]. It is likely that the pacemaker clusters that dominate at sympathetic and parasympathetic tone are mediated majorly by the Ca^2+^ clock which is disrupted in CPVT patients by mutations in Ca^2+^ handling proteins.

It has been known for over a century that pacemaker cells are widely distributed throughout the entire intercaval region located between the superior and inferior vena cava and between the crista terminalis and intra-atrial septum [105,106,107]. Canine and human studies [61,108,109,110,111,112] in which potentials have been recorded from multiple electrodes simultaneously have revealed an extensive distributed system of atrial pacemakers (the atrial pacemaker complex), which includes but extends well beyond an anatomically defined SAN. In some circumstances, when the SAN is sick (i.e., sinus node dysfunction or sick sinus syndrome) or temporally suppressed (for instance, during extensive parasympathetic stimulation), automaticity could be observed from other clusters of subsidiary atrial pacemakers located within the distributed atrial pacemaker complex [49,113]. These cells have been characterized to express both pacemaker marker HCN4 and non-pacemaker marker Cx43 [20,113,114]. Though these subsidiary pacemaker clusters can provide a relatively regular rhythm, they are characterized by a slower resting heart, slower exertional heart rates, a prolonged post-pacing recovery time (a parameter similar to SAN recovery time but for non-SAN pacemakers), and an increased beat-to-beat heart rate variability [48,49,114,115,116]. Furthermore, the electrical activity of this subsidiary pacemakers is more akin to that of the SAN than to the surrounding atrial muscle; the subsidiary pacemaker action potential exhibits prominent diastolic depolarization and a significantly lower maximum diastolic potential, take-off potential, overshoot, rate of rise, and amplitude than typical atrial muscle [117]. Finally, while being bradycardic in general, subsidiary atrial pacemakers can contribute to the development of atrial tachycardia [118,119,120].

## 6. Conclusions

Here, we summarize the experimental evidence supporting the presence of hierarchical pacemaker clustering within the SAN. The studies discussed highlight the importance of these intranodal pacemaker clusters in a complex and dynamic regulation of the heart rhythm in response to various interventions. Importantly, we show that SAN clusters may differentially rely on the components of the coupled clock pacemaker system which, in turn, determines their sensitivity to both physiological and pathophysiological perturbations as well as genetic mutations. The latter may critically contribute to the development of the sick sinus syndrome and its manifestation under certain conditions that affect certain pacemaker clusters. These findings further highlight a complex nature of the SAN pacemaker complex and can introduce novel targets that could be used for the treatment of various SAN pathologies.
